# Regression discontinuity analysis for pharmacovigilance: statin example reflected trial findings showing little evidence of harm

**DOI:** 10.1016/j.jclinepi.2021.10.003

**Published:** 2022-01

**Authors:** Lauren Scott, Maria Theresa Redaniel, Matthew Booker, Rupert A. Payne, Kate Tilling

**Affiliations:** a National Institute for Health Research Applied Research Collaboration West, University Hospitals Bristol and Weston NHS Foundation Trust, 9th Floor, Whitefriars, Lewins Mead, Bristol, BS1 2NT, UK; b Population Health Sciences, Bristol Medical School, University of Bristol, Canynge Hall, 39 Whatley Road, Bristol, BS8 2PS, UK; c Centre for Academic Primary Care, Bristol Medical School, Canynge Hall, University of Bristol, 39 Whatley Road, Bristol, BS8 2PS, UK; d MRC Integrative Epidemiology Unit, Bristol Medical School, Oakfield House, Oakfield Grove, Bristol, BS8 2BN, UK

**Keywords:** Regression discontinuity analysis, Statins, Cardiovascular disease, QRISK score, Epidemiology, Health services research

## Abstract

**Objectives:**

The study aims to explore the use of regression discontinuity analysis (RDA) to examine effects of prescription of statins on total cholesterol and adverse outcomes (type 2 diabetes, rhabdomyolysis and myopathy, myalgia and myositis, liver disease, CVD, and mortality).

**Study Design and Setting:**

We conducted a prospective cohort study using the Clinical Practice Research Datalink including patients with QRISK scores of 10 to 30 in 2010 to 2013 who were last followed-up in October 2016. Comparing patients with QRISK≥20 and QRISK<20, we explored RDA assumptions, provided proof of concept analyses (total cholesterol as outcome), and investigated the effect of statins prescription on adverse outcomes.

**Result:**

RDA confirmed statin prescription reduced total cholesterol (Mean difference (MD) -1.33 mmol/L, 95%Confidence Interval (CI) -1.93 to -0.73). RDA provided little evidence for adverse effects on diabetes, myalgia and myositis, liver disease, CVD, or mortality. The RDA analysis findings are similar to RCT results. Findings from non-RDA analysis agree with published observational studies.

**Conclusion:**

RDA can be used with large routine clinical datasets to provide evidence on effects of medications which are prescribed according to a threshold. Testable RDA assumptions were satisfied, but confidence intervals were wide, partly due to the low compliance with the prescribing threshold.

## Highlights

• RDA can be used with routine healthcare data, resulting in findings similar to Randomized controlled trials.
• Application of regression discontinuity analysis (RDA) in epidemiology should include examination of the assumptions.
• RDA should employ sensitivity analyses and negative control analyses.
• RDA should carefully consider choice of model and bandwidth.

**What is new?**

• RDA can be used with routine healthcare data, resulting in findings similar to RCTs.
• Application of RDA in epidemiology should include examination of the assumptions.
• RDA should employ sensitivity analyses and negative control analyses.
• RDA should carefully consider choice of model and bandwidth.

## 1 Introduction

Randomized controlled trials (RCTs) are top of the hierarchy of evidence [1], but may not be generalizable (strict eligibility criteria) or possible (ethical issues, low power for rare conditions), and are expensive, time consuming, and often short follow-up [2,3]. Using observational data addresses some issues, but is subject to unmeasured confounding.

Regression discontinuity analysis (RDA) is a quasiexperimental method that can be used to estimate treatment effects where provision depends on predefined threshold on a continuous scale. Scores just above or below this threshold are analogous to random assignment to treatment or control group [4]. If assignment is deterministic, that is, everyone one side of the threshold will receive treatment and everyone the other side will not, a “sharp RDA” can be applied. If the assignment is probabilistic, that is, more likely to receive treatment if on one side of the threshold than the other, then "fuzzy RDA" can be used [5]. Both assignment rules infer causality, without bias by confounding [4,6].

Cardiovascular disease (CVD) is a common cause of mortality in the UK, accounting for approximately 25% of deaths [7,8]. High cholesterol levels are a factor for CVD [9]. Statins are prescribed to reduce cholesterol [10], but evidence around safety is conflicting. Observational studies identified adverse effects including type 2 diabetes, rhabdomyolysis, myopathy, myalgia, myositis, and liver disease [11], [12], [13]. However, two systematic reviews of RCTs reported only small increases in diabetes risk and no differences in risks of other outcomes [14,15].

The QRISK/QRISK2 cardiovascular risk score (QRISK score hereafter) uses factors including cholesterol, sex, and age to predict CVD event risk in the next 10 years [16,17], for example, a score of 20 indicates 10-year risk of 20%. Current guidelines recommend that QRISK is used to assess CVD risk for patients up 84 years, with those over 40 years risk reviewed on an ongoing basis [20]. Between 2008 and 2014, the National Institute for Health and Clinical Excellence (NICE) recommended general practitioners (GPs) prescribe statins to patients aged 18 and above with QRISK score 20 or above [17], [18], [19], [20] (since amended to 10). This score can be used as assignment variable to study statins using RDA [6,[21], [22], [23], [24]].

The study aims to explore the use of RDA to examine side effects of medications. We present methodological considerations using statins prescription as an example. RDA analyses were split into: (1) exploration of RDA assumptions; (2) proof of concept analyses with total cholesterol as outcome; (3) investigation of the effect of statins on the adverse outcomes; (4) sensitivity analyses.

## 2 Methods

### 2.1 Regression discontinuity analysis

Clinicians consider many factors when prescribing statins so assignment rule is likely probabilistic (rather than deterministic). “Fuzzy” RDA was applied using two-stage least squares instrumental variable (IV) analysis [5].

The following four key assumptions need to be satisfied [25]:

1. There is a discontinuity in the probability of receiving the exposure (statin prescription) at the threshold
2. Individual values of the assignment variable (QRISK score) are not manipulated
3. Exposure groups are exchangeable at the threshold
4. The outcome probability would be continuous at the threshold in the absence of the exposure (statin prescription)

Bandwidth around the threshold (how close to the threshold scores should be for inclusion in analysis) is important for Assumption 3. A narrower bandwidth makes this assumption more plausible, but leads to fewer participants and lower power.

### 2.2 Data sources

This study used the Clinical Practice Research Datalink (CPRD), which includes records of approximately 19 million patients from over 900 UK primary-care practices [26]. CPRD was linked to Hospital Episode Statistics-admitted patient care (HES-APC) and Office for National Statistics (ONS) mortality data.

### 2.3 Study population

Patients with a 10≤QRISK<30 read code in CPRD between 2010 and 2013, 40 to 84 years old, had not been prescribed statins or diagnosed with type 2 diabetes or CVD prior to their QRISK score, were included. Current NICE guidelines advocate against the use of QRISK in the presence of cardiovascular disease and type 1 diabetes, and the use of statins for people with any diabetes to prevent CVD [20].

Patient look back period was from time of registration with the practice or when the practice data was deemed of up-to-standard research quality (mean: 18.9 years, SD:13.7, Appendix B). Patients were followed-up from the date of the first QRISK score (index date) or 92 days after the index date (for proof of concept analysis) to the earliest of: date of the outcome, patient transfer-out from practice, patient's death, practice last date of data collection or October 2016 (study end date).

### 2.4 Study exposure

For patients with more than one QRISK measurement, only the first score was used. The discontinuity variable was derived by dichotomizing the QRISK score at 20 (QRISK≥20 or QRISK<20, coded as 1 and 0 respectively).

Statins prescriptions (one or more) within 60 days of the index date were identified using CPRD product codes (see Appendix 1), and coded as binary variable (0 = no statins, 1 = yes statins).

### 2.5 Study variables

For proof of concept, we investigated the cholesterol reduction from statins. Statin prescriptions within 60 days of the index date was used as exposure. We then allowed for a month after this period (61–91 days) to allow time for patients to take the prescribed statins. The first total cholesterol value between 92 and 456 days after the index date was used in the analysis to allow for a 12-month follow-up period (Appendix B).

Outcomes examined using RDA were type 2 diabetes, myalgia and myositis, rhabdomyolysis and toxic myopathies, liver disease, CVD and mortality. Mortality was derived from death dates within CPRD and/or ONS data; all other outcomes were derived from read codes (CPRD), ICD-10 codes (HES-APC) or ONS cause of death data (see Appendix 1). CVD events included strokes, TIAs, and myocardial infarctions. For each outcome, only the occurrence of the first event after index date was used in the analysis. This is in line with an intention-to-treat (ITT) analysis in RCTs where follow-up starts at time of randomization (i.e., date of the QRISK score in this analysis). From a clinical perspective, side effects could occur within a short interval after exposure to statins.

To identify adhering practices, that is, where statins were more likely to be prescribed to patients with QRISK≥20 than with QRISK<20, we fitted linear mixed model with statins prescription (0 = no statins, 1 = yes statins) as outcome; continuous QRISK score, binary discontinuity, and the interaction between these as fixed effects; and binary discontinuity nested within categorical GP practice as a random effect. Adhering practices were those whose random effect for discontinuity was positive.

Negative control analysis was performed using hospitalization for any injury or poisoning events during the study period as the outcome, using the ICD-10 codes in the HES data (see Appendix A).

Continuous data are summarized using means and standard deviations (SDs), or medians and interquartile ranges (IQRs) for skewed distributions. Binary data are summarized using counts and percentages. We examined mean differences (MDs) for continuous outcomes (total cholesterol), and risk difference (RDs) for binary outcomes (CVD, diabetes, myalgia, rhabdomyolysis, liver disease and mortality).

### 2.6 Statistical methods

QRISK scores were rounded down into groups (i.e., 19.00–19.99, 20.00–20.99, etc) for summaries. To explore the first RDA assumption, the proportion of patients prescribed statins within each QRISK group were plotted for all included patients and the subset who attended adhering practices. The F-statistics for instrument strength were explored, based on the primary mortality model (as this model had no missing data). For assumption two, the frequency of patients in each QRISK group were plotted to check for unexpected changes either side of the threshold and tested using a regression discontinuity manipulation test (Stata *rddensity*) [27,28]. For assumption three, the proportion of patients within each QRISK group were plotted by variables that could plausibly affect the probability of being prescribed statins (sex, age, length of follow-up, prescribing contraindication to statins; Appendix A) and total cholesterol on or prior to index date. Assumption four cannot be tested directly; however, injuries or poisoning requiring hospitalization was explored as a negative control outcome. We have no reason to believe injuries or poisoning to not have a continuous probability discontinuity at the threshold (QRISK = 20). Injuries or poisoning is subject to sociodemographic confounding, as statin prescription is, supporting its use as a negative control outcome. Age and gender could be associated with injuries and have been controlled for in the analysis. All assumptions were tested using QRISK = 10 and QRISK = 30 as negative control exposures (NCE).

As a proof of concept, known reduction in cholesterol from statins [15] was investigated using RDA. Firstly, IV analysis (Stata *ivregress*) was used assuming a linear effect of QRISK on total cholesterol either side of the threshold. Secondly, additional quadratic effects were allowed either side of the threshold. Finally, multiple imputation [29,30] of total cholesterol scores for patients without cholesterol measured 3 to 15 months post-index was explored: 20 imputed datasets were generated (Stata *mi impute)* with the following predictors: age, total cholesterol prior to/on index date, total cholesterol 3 to 15 months post-index date, index year, QRISK score, statins, and an interaction term QRISK x statins; the imputation models were stratified by sex and discontinuity. The most recent total cholesterol value recorded in the patient records prior to or on the date of the QRISK score and within the data up-to-standard date were used in the imputation. The average look-back period prior to index date is 175 days with a median of 13 days (IQR: 39 and 6 days). Multiple imputation relies on the assumption that missingness is at random conditional on the variables in the imputation model and that the imputation model is correctly specified; these assumptions are not testable given the observed data. For all three approaches, we investigated the effect of different bandwidths on either side of the threshold. In all models, the covariates were: continuous QRISK scores on either side of the threshold (either linear or linear and quadratic) binary statins and sex variables, and a categorical age variable; the instrument was the binary discontinuity variable. All remaining analyses were done using the 10 to 30 bandwidth,

To explore the effect of statins on outcomes, we used linear IV regression with bandwidth 10 to 30 to estimate MD or RD. We assumed that if an outcome was not recorded it had not occurred. We also investigated sensitivity analyses using bandwidth 15 to 25 and additional quadratic terms as described above. We have adjusted for age and sex in all outcome models.

We used linear regression to estimate treatment effects for comparison with RDA. These models included the full bandwidth 10 to 30 in adhering practices, and were adjusted for linear QRISK score, age, and sex. The exposure was whether each person had been prescribed statins within the 60 days after index date. Based on reported incidence rates for our outcomes [31], [32], [33], [34], [35], [36], ranging from 0.01% (for myopathy and rhabdomyolosis) to 20% (myalgia), assuming 0.05 significance level with 80% power, we have calculated, using non-RDA methods for comparing two proportions or means, that we will be able to detect effect sizes ranging from 0.1% to 20% in exposed group.

Sensitivity analyses removed outcomes occurring within two weeks and within 60 days of the index date, and included all practices. We did not consider death as a competing risk for other outcomes as the risk of death in this sample is low (2.4%).

Stata 15.1 [37] was used for all analyses**.**

## 3 Results

From 2010 to 2013, 87,381 patients met the eligibility criteria; 31,649 had 10≤QRISK<30 and made up the study population. Median age was 65 years (IQR 60 to 69),44.1% were female, 27.3% had QRISK≥20, 8.7% were prescribed statins within 60 days, median follow-up period is 3 years (IQR: 1.9, 3.9), and 11,758 attended adhering practices (Table 1). There was no change in the distributions (apart from statin prescription) when the sample is limited to adhering practices. There were no rhabdomyolysis or toxic myopathies events.

**Table 1:** Patient characteristics and outcomes in all and adhering practices

All study patients	10≤ QRISK <20 (*n* = 23,015)		20≤ QRISK <30 (*n* = 8,634)		Overall (*n* = 31,649)	
	n	%	n	%	n	**%**
**Patient characteristics**						
Age (y; median, IQR)	63	(58, 67)	68	(64, 72)	65	(60, 69)
Female	11,132/23,015	48.4%	2,820/8,634	32.7%	13,952/31,649	44.1%
Prescribed statins	936/23015	4.1%	1,824/8,634	21.1%	2,760/31649	8.7%
Total cholesterol (prior to index date, mmol/l; mean SD))	5.7	1.0	5.7	1.0	5.7	1.0
Contraindication	5,657/23,015	24.6	2,370/8,634	27.4	8,027/31,649	25.4
Follow-up duration (years; median, IQR)	3.0	(1.9, 3.8)	3.1	(2.0, 3.9)	3.0	(1.9, 3.9)
**Outcomes**						
Total cholesterol (mmol/l; mean, SD)^a^	5.6	1.0	5.2	1.1	5.5	1.1
Type2 diabetes	529/23,015	2.3%	332/8,634	3.9%	861/31,649	2.7%
Myalgia and myositis	144/22,307	0.7%	75/8,396	0.9%	219/30,703	0.7%
Rhabdomyolysis and toxic myopathies	2/23,009	0.0%	2/8,631	0.0%	4/31,640	0.0%
Liver disease	82/22,898	0.4%	46/8,585	0.5%	128/31,483	0.4%
Cardiovascular disease	436/23,015	1.9%	282/8,634	3.3%	718/31,649	2.3%
Mortality	408/23,015	1.8%	313/8,634	3.6%	721/31,649	2.3%

Adhering practices only	10≤ QRISK <20 (*n* = 8,443)		20≤ QRISK <30 (*n* = 3,315)		Overall (*n* = 11,758)	
	n	%	n	%	n	%
**Patient characteristics**						
Age (years; median, IQR)	63	(57, 66)	68	(63, 72)	64.0	(59, 68)
Female	4,099/8,443	48.5%	1,152/3,315	34.8%	5,251/11,758	44.7%
Prescribed statins	510/8,443	6.0%	1,159/3,315	35.0%	1,669/11,758	14.2%
Total cholesterol (prior to index date, mmol/l; mean SD))	5.8	1.0	5.8	1.0	5.8	1.0
Contraindication	5,657/8,443	24.6	2,370/3,315	27.4	8,027/11,758	25.4
Follow-up duration (years; median, IQR)	3.1	(2.1, 4.0)	3.2	(2.2, 4.1)	3.2	(2.1, 4.0)
**Outcomes**						
Total cholesterol (mmol/l; mean, SD)^a^	5.6	1.0	5.1	1.1	5.4	1.1
Type2 diabetes	204/8,443	2.4%	145/3,315	4.4%	349/11,758	3.0%
Myalgia and myositis	61/8,155	0.7%	37/3,222	1.1%	98/11,377	0.9%
Rhabdomyolysis and toxic myopathies	0/8,438	0.0%	0/3,313	0.0%	0/11,751	0.0%
Liver disease	24/8,404	0.3%	9/3,305	0.3%	33/11,709	0.3%
Cardiovascular disease	153/8,443	1.8%	110/3,315	3.3%	263/11,758	2.2%
Mortality	155/8,443	1.8%	122/3,315	3.7%	277/11,758	2.4%

a Data missing for 5,886/8,443 patients in the 10≤QRISK<20 group and 1,863/3,315 in the 20≤QRISK<30 group.

### 3.1 Testing RDA assumptions

#### 3.1.1 Assumption 1: Discontinuity in the probability of the exposure (statins prescription) at the threshold

**Fig. 1:**
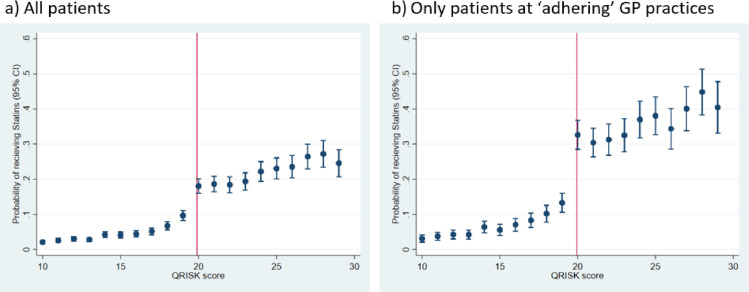
Assumption 1: Discontinuity in the probability of being prescribed statins at the QRISK score threshold.

A discontinuity at the threshold of QRISK = 20 was observed: 9.7% of patients with QRISK = 19 were prescribed statins compared to 18.1% of patients with QRISK = 20 (Fig. 1A; discontinuity estimate 9.0%, 95% CI 7.8–10.3, *P*< 0.001). In the subset of patients attending adhering GP practices, the discontinuity was larger: 13.3% of patients with QRISK = 19 were prescribed statins compared to 32.7% of patients with QRISK = 20 (Fig. 1B; discontinuity estimate 18.0%, 95% CI 15.6–20.5, *P* < 0.001). The instrument strength F-statistic was higher in this adhering subset than all patients (205.9 vs. 193.0). Given that the checks indicate the discontinuity estimate is a stronger instrument in the subset, all remaining checks and analyses were carried out on the adhering subpopulation.

#### 3.1.2 Assumption 2: Individual values of the assignment variable (QRISK score) are not manipulated

**Fig. 2:**
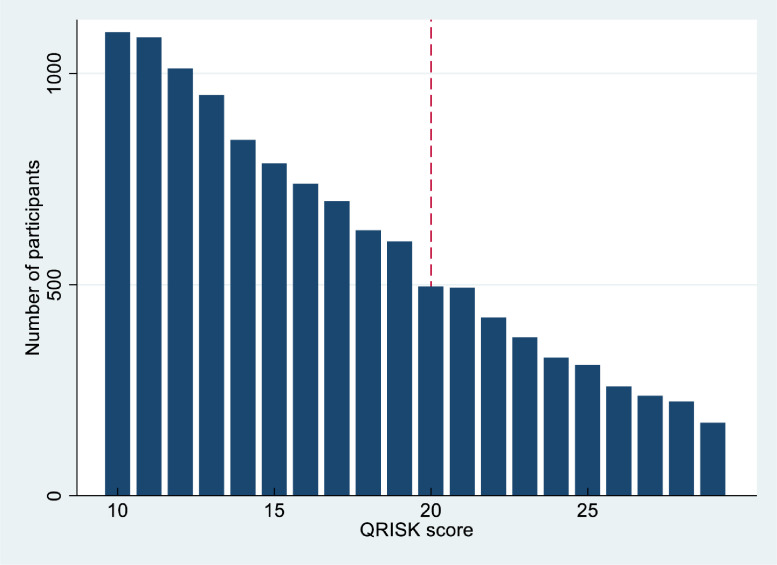
Assumption 2: Individual values of the assignment variable are not manipulated – histogram of QRISK scores.

It would be difficult for a patient to manipulate their QRISK score, but it would, in theory, be possible for a GP to manipulate it by rounding for patients very close to the threshold. Whilst the number of people with QRISK score at 20 is slightly lower than expected (Fig. 2), there was no substantial peak or trough around the threshold (regression discontinuity manipulation test *P* = 0.47), suggesting assumption 2 is plausible.

#### 3.1.3 Assumption 3: Exposure groups are exchangeable at the threshold

**Fig. 3:**
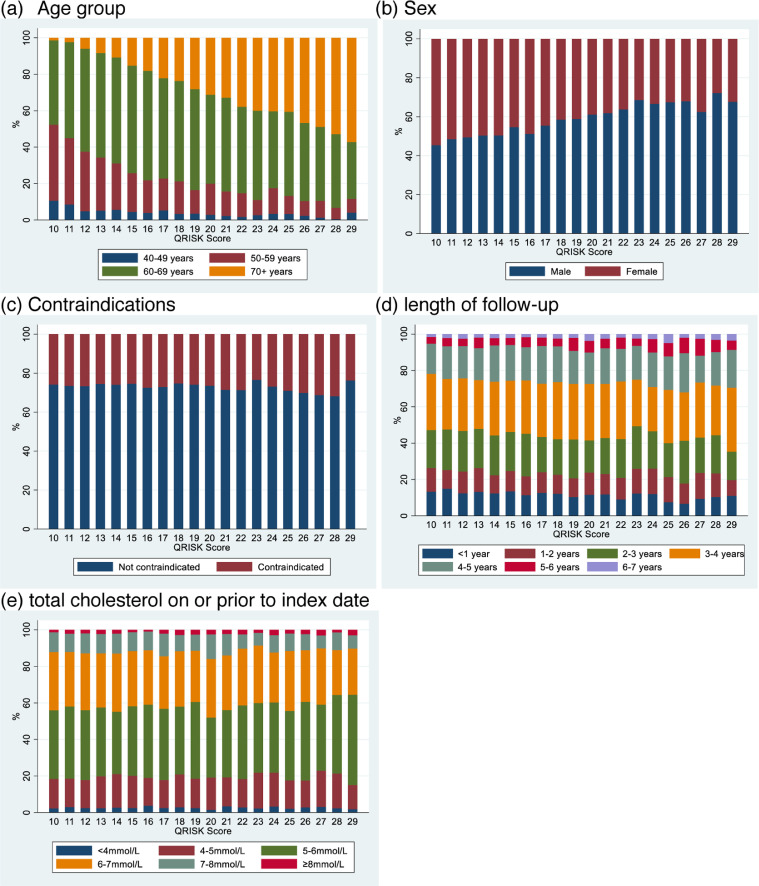
Assumption 3: Exposure groups are exchangeable at the cut-off-distributions of key measured confounders by QRISK score in the adhering population.

Figs. 3A–3E show the distributions of several characteristics by QRISK score. Older patients and male patients were more likely to have higher QRISK scores. This was expected as age and sex are both part of the QRISK calculation; all analyses adjust for these covariates. A patient's follow-up time, their total cholesterol score, and whether they were contraindicated for statins did not substantially differ by QRISK score. For all characteristics, there was no clear distribution change either side of the threshold, meeting Assumption 3. However, the possibility of unmeasured non-exchangeability cannot be examined.

#### 3.1.4 Assumption 4: The outcome would be continuous at the threshold in the absence of the exposure (statins prescription)

This assumption is not directly testable. However, a negative control linear regression analysis with the outcome of any hospital admission for injuries or poisoning we found no discontinuity in injury rate at the threshold (discontinuity 0.19%, 95% CI -1.47 to 1.86, *P*= 0.82). This suggested that assumption 4 is plausible.

Negative control exposures using QRISK = 10 or QRISK = 30 showed no evidence of effects on total cholesterol: MD: 7.19mmol/l, 95% CI: -0.17 to 14.56 (QRISK = 10) and MD: -11.65, 95% CI: -81.56 to 58.26 (QRISK = 30).

### 3.2 RDA proof of concept total cholesterol findings

Thirty-four percent (4,009/11,758) of patients had a total cholesterol value recorded 3 to 15 months postindex. The first total cholesterol value measured between 92 and 456 days after the index date was used to allow patients to have taken their statins for at least a month and to ensure that follow-up period did not allow for high rates of intervening treatment (15.2% of patients [3,496/23,015] who initially had a QRISK score<20 had it remeasured within 15months, 2.1% [484/23,015] had a QRISK score>=20 during this period, and 139 of these patients were then subsequently prescribed statins). Total cholesterol was lower for patients with QRISK≥20 than QRISK<20 (5.1 mmol/L [SD 1.1] vs. 5.6 [SD 1.0]).

IV analyses showed that statins reduce total cholesterol (Table 2; MD: -1.33 mmol/L, 95% CI: -1.93 to -0.73). Sensitivity analysis using different bandwidths and including quadratic terms (Appendix C) showed similar results, with IV estimates between -0.77 mmol/L (95% CI -1.86 to 0.32) and -1.62 mmol/L (95% CI -2.41 to -0.83). These findings are similar to those seen in RCTs [15]. The conclusions drawn from the differing model specifications and bandwidths are similar (Appendix C and D).

### 3.3 Adverse events

**Fig. 4:**
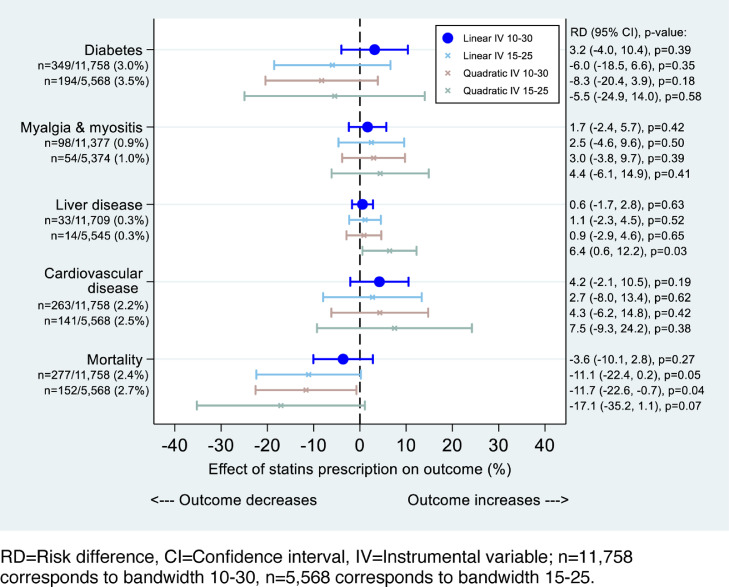
RDA of all outcomes. RD = Risk difference, CI = confidence interval, IV = Instumental variable; n = 11,758 corresponds to bandwidth 10–30, *n* = 5,568 corresponds to bandwidth 15-25.

There are proportionally more events in patients with QRISK≥20 than QRISK<20 for most outcomes, except liver diseases (same in the two groups) (Table 1). Statins had no effect on type2 diabetes, myalgia and myositis, and liver disease (Table 2, Fig. 4). Sensitivity analyses adjusting bandwidth and including quadratic terms did not improve model fit nor change the conclusions for diabetes or myalgia and myositis (Appendix C). For liver disease, the effect estimates remained very similar for most analyses, but the quadratic IV analysis with bandwidth 15 to 25 suggested an increase in liver disease with statin prescription (RD 6.4, 95% CI 0.6–12.2, *P*= 0.03). We found some evidence of an increase in CVD (RD 4.2, 95% CI -2.1 to 10.5, *P*= 0.19), and a decrease in mortality (RD -3.6, 95% CI -10.1 to 2.8, *P*= 0.27) with statin prescription albeit with wide confidence intervals. The sensitivity analyses for mortality found some evidence for a protective effect of statins (Fig. 4). Further sensitivity analyses removing outcomes occurring within two weeks or within 60 days of the index date and including all practices did not change the results (Appendix E and F).

**Table 2:** Summary of estimated effect of statin prescription on cholesterol and adverse outcomes using RDA, unadjusted and adjusted and linear regression

Outcome	RDA				Linear regression*	
	Unadjusted		Adjusted*			
	MD/RD	95% CI	MD/RD	95% CI	MD/RD	95% CI
Total cholesterol	-1.38	-1.99, -0.76	-1.33	-1.93, -0.73	-0.86	-0.93, -0.78
Type2 diabetes	3.71	-3.43, 10.85	3.18	-4.0, 10.37	2.07	1.12, 3.01
Myalgia and myositis	1.55	-2.46, 5.55	1.67	-2.38, 5.71	1.22	0.69, 1.74
Liver disease	0.50	-1.73, 2.73	0.56	-1.69, 2.81	-0.19	-0.48, 0.11
Cardiovascular disease	4.33	-1.92, 10.58	4.21	-2.09, 10.51	0.65	-0.17, 1.47
Mortality	-3.79	-10.17, 2.59	-3.63	-10.06, 2.80	-1.71	-2.55, -0.87

⁎ Adjusted for age and sex.

### 3.4 Observational analyses

In adhering practices, the estimated effects of statin prescription from observational analyses were similar to those from RDA, but closer to the null.

## 4 Discussion

### 4.1 Summary of results

Our study suggested that RDA was appropriate; we found an effect of statins on total cholesterol similar to that seen in RCTs [15]. Statins have little effect on any adverse outcome and a possible decrease in mortality. Contrary to expectations, we found some very weak evidence that statins increased CVD. This might be due to the exclusion of patients with QRISK≥30, who are at higher risk of CVD and may benefit most from statins, or unmeasured confounding due to wide bandwidth. Sensitivity analyses highlighted that conclusions were not sensitive to model specification and bandwidth. The observational (non-RDA) findings mostly agreed with other observational studies [11], [12], [13].

### 4.2 Strengths & limitations

We showed that RDA assumptions were plausible when exploring statin side effects. The use of routinely collected data meant participation was not biased by eligibility restrictions applied to RCTs, and the study population was large (11,758 patients in our dataset; 155–9014 patients in similar RCTs [14]). We approximately replicated results from RCTs for the effects of statins on total cholesterol [14]. The use of RDA methodology can overcome unmeasured confounding, providing the RDA assumptions were satisfied.

The choice of bandwidth was a pragmatic decision balancing maximizing the power of the study (by choosing the largest bandwidth) with maximizing the plausibility of the exchangeability assumption (i.e., assumption of no unmeasured confounding) by choosing the smallest bandwidth. The chosen bandwidth accounts for GP prescribing behavior. At the study time period, QRISK score of 10 was considered low risk and we expect few patients below this threshold to have been prescribed statins. Contrastingly, a QRISK score of 30 was considered high risk and we expect these patients to have been prescribed statins.

Our results were based on patients with a QRISK score of 10 to 30 who are registered with adhering GP practices, which could have affected generalizability. Sensitivity analysis testing the first RDA assumption showed that QRISK discontinuity is a stronger instrument for statin treatment by including only adhering practices. RDA estimates local average treatment effects, that is, effects of statin prescription on people with a QRISK close to the cut-off.

The decision on the timings regarding the 0 to 60 days for statin prescription and the 92 to 456 days follow-up for the proof of concept analysis were made pragmatically, accounting for the balance between longer follow-up and confounding. Longer follow-up will result in more outcome events but increases the chances of competing risks. From a clinical perspective, most important side effects will occur within a year. We do not believe these decisions could have biased the results due to the nature of RDA, because length of follow-up was balanced between those with QRISK<20 and >20.

Missingness was also a limitation. Only 75% of patients had linked HES data, which could have resulted in underreporting of outcomes, but is likely non-differential. Change in status during the follow-up period, that is, from low to high QRISK scores (no statins to statins) and vice versa, was not accounted for. A potential limitation of using the read coded QRISK scores (rather than calculating from the components) is that GPs may be more likely to record scores for patients who they prescribe statins to; however, we did not find this to be the case, with few patients prescribed statins.

The effect tested is being prescribed statins because of a QRISK score just above or below the threshold. Further prescriptions during the follow-up period for those with an initial QRISK below 20 would tend to bias effect estimates towards the null. This is analogous to an ITT analysis of an RCT with noncompliance in the intervention arm and the ability to take treatment upon request in the control arm.

A limitation of using RDA for exploring side effects is the exclusion of low (<10) and high (≥30) QRISK values, where the side effects (or benefits) are likely more apparent. Further, we were unable to account for statins dose; dose is likely to be higher for patients with higher scores, and side effects (and benefits) from these higher doses may have been missed.

Even when only including adhering practices, the discontinuity in prescribing practice at the threshold was not large. However, the null results of the negative control outcome and exposure supports the assumption that the outcomes are continuous in the absence of statin prescription. We are assuming that some of the same factors causing high cholesterol and other outcomes would also cause injuries. Hence, if the discontinuity is caused by another factor, we would expect that factor also to cause a discontinuity in the injury rates.

We do not have information on all variables that could have influenced model precision (e.g., GP experience, Quality Outcomes Framework indicators). While inclusion of these variables would strengthen the instrument, their exclusion should not cause bias. We were unable to examine effect modification by deprivation, gender or age, due to small numbers of events. Side-effects could also vary by type of statin prescribed – however here, aside from low power for stratified analysis by statin type, we do not have an instrument for prescription of a specific statin over another. The calculation for sample size was for the observational analysis using linear regression – larger numbers are required for IV analyses, depending on the strength of the instrument.

Lastly, we only captured prescriptions, not whether patients took the statins. We could not ascertain whether the absence of effects for CVD and mortality could be due to patients not taking prescribed medications. Nonadherence to medication would reduce the number of side effects in the group prescribed statins. This is the same issue as with the ITT analysis of an RCT with noncompliance in the treatment group, and would result in underestimate of the side-effects.

### 4.3 Comparison with other studies

RDA is becoming more popular in healthcare research [38]. There are a few recently published studies which describe RDA using statins as an example [6,[21], [22], [23], [24]], but these mostly used simulated data. Two studies [21,23] used UK primary care data to provide a worked example with LDL cholesterol, but had very selective samples (e.g., only male, non-smokers aged 50–70) and did not investigate other outcomes. To our knowledge, this is the first study to investigate adverse effects of statins using RDA methods.

Many RCTs investigated the effect of statins on cholesterol and CVD. Two systematic reviews [14,15] concluded that diabetes was the only adverse event, but that the absolute difference between diabetes rates in the statins group vs. the placebo group were small (e.g., absolute difference of 0.4% [15]). Our study found larger point estimates but also wider 95% confidence intervals spanning zero. Both reviews also found that rhabdomyolysis was extremely rare (e.g., only 3 of 19,410 [0.02%] [15]).

### 4.4 Implications for research & practice

We demonstrated that RDA can be used with routine healthcare data, resulting in findings similar to RCTs. Inclusion of sensitivity analyses in outcome studies using RDA are important. Potential instruments for RDA include age-thresholds [25], time-thresholds [39] and treatment thresholds [40]. Application of RDA in epidemiology should include examination of the assumptions, sensitivity analyses and negative control analyses.

## 5 Conclusions

RDA can be used with large routine clinical datasets to provide evidence on effects of medications which are prescribed according to a threshold.

## Appendix Supplementary materials

Image, application 1

Image, application 2

Image, application 3

Image, application 4

Image, application 5

Image, application 6
