# Supplementary material for: Regression discontinuity analysis for pharmacovigilance: statin example reflected trial findings showing little evidence of harm
Source: J Clin Epidemiol. 2022 Jan;141:121–31. doi: 10.1016/j.jclinepi.2021.10.003 (PMC8982642; doi:10.1016/j.jclinepi.2021.10.003)
Supplement: Supplementary file 1 [file mmc1.docx]

# **Appendix A – Code lists**

| **Name** | **List** |
| --- | --- |
| Statins |  |
| Contraindicated drugs |  |
| CVD |  |
| Diabetes |  |
| Rhabdomyolosis and myopathies |  |
| Myaliga and myositis |  |
| Liver diseases |  |
| Injury and poisoning |  |
