# Supplementary material for: Regression discontinuity analysis for pharmacovigilance: statin example reflected trial findings showing little evidence of harm
Source: J Clin Epidemiol. 2022 Jan;141:121–31. doi: 10.1016/j.jclinepi.2021.10.003 (PMC8982642; doi:10.1016/j.jclinepi.2021.10.003)
Supplement: Supplementary file 2 [file mmc2.docx]

# **Appendix B. Study periods**

**For proof of concept analysis using total cholesterol as outcome**

|  |  |  |  |  |  |  |  |  |  |  |  |  |  |  |
| --- | --- | --- | --- | --- | --- | --- | --- | --- | --- | --- | --- | --- | --- | --- |
|  | Look-back  period | | | Exposure assessment  period | | |  | | | Follow-up | | |  |  |
|  |  |  |  |  |  |  |  |  |  |  |  |  |  |  |
|  |  |  | 0 | |  | 60 | |  | 92 | |  | 456 days | |  |
| Time of practice registration / practice data deemed up-to-standard | |  | QRISK score (Index date) | |  | end of exposure period | |  | start of follow-up period | |  | end of follow-up period | |  |

**Regression Discontinuity Analysis for all other outcomes**

|  |  |  |  |  |  |  |  |  |  |  |  |  |  |  |
| --- | --- | --- | --- | --- | --- | --- | --- | --- | --- | --- | --- | --- | --- | --- |
|  | Look-back  period | | | Exposure assessment  period | | |  | | |  | | |  |  |
|  |  |  |  | Follow-up | | | | | | | | |  |  |
|  |  |  |  |  |  |  |  | | | | | |  |  |
|  |  |  | 0 | |  | 60 | |  |  | |  | 456 days | |  |
| Time of practice registration / practice data deemed up-to-standard | |  | QRISK score (Index date)  and start of follow-up period | |  | end of exposure period | |  |  | |  | end of follow-up period | |  |
