# Supplementary material for: Regression discontinuity analysis for pharmacovigilance: statin example reflected trial findings showing little evidence of harm
Source: J Clin Epidemiol. 2022 Jan;141:121–31. doi: 10.1016/j.jclinepi.2021.10.003 (PMC8982642; doi:10.1016/j.jclinepi.2021.10.003)
Supplement: Supplementary file 3 [file mmc3.docx]

**Appendix C. RDA of total cholesterol (proof of concept), using different bandwidths and linear and quadratic terms**

MD=Mean difference, CI=Confidence interval, IV=Instrumental variable, MI=Multiple imputation
