# Supplementary material for: Regression discontinuity analysis for pharmacovigilance: statin example reflected trial findings showing little evidence of harm
Source: J Clin Epidemiol. 2022 Jan;141:121–31. doi: 10.1016/j.jclinepi.2021.10.003 (PMC8982642; doi:10.1016/j.jclinepi.2021.10.003)
Supplement: Supplementary file 4 [file mmc4.docx]

**Appendix D: Patient characteristics and outcomes for bandwidth 15-25, adhering practices only.**

| **Variable** | **15≤ QRISK <20**  **(n=3,455)** | | **20≤ QRISK <25**  **(n=2,113)** | | **Overall**  **(n=5,568)** | |
| --- | --- | --- | --- | --- | --- | --- |
|  | **n** | **%** | **n** | **%** | **n** | **%** |
| **Patient characteristics** | | | | | | |
| Age (years; median, IQR) | 63 | (60, 69) | 67 | (62, 71) | 66 | (61, 70) |
| Female | 1,538/3,455 | 44.5% | 761/2,113 | 36.0% | 2,299/5,568 | 41.3% |
| Prescribed statins | 298/3,455 | 8.6% | 687/2,113 | 32.5% | 985/5,568 | 17.7% |
| Total cholesterol (prior to index date,  mmol/l; mean SD)) | 5.8 | 1.0 | 5.8 | 1.0 | 5.8 | 1.0 |
| Contraindication | 907/3,455 | 26.2 | 569/2,113 | 26.9 | 1,476/5,568 | 26.5 |
| Follow-up duration (years; median, IQR) | 3.2 | (2.1, 4.0) | 3.2 | (2.1, 4.0) | 3.2 | (2.1, 4.0) |
| **Outcomes** | | | | | | |
| Total cholesterol (mmol/l; mean, SD) ^a^ | 5.5 | 1.0 | 5.1 | 1.1 | 5.3 | 1.1 |
| Type2 diabetes | 103/3,455 | 3.0% | 91/2,113 | 4.3% | 194/5,568 | 3.5% |
| Myalgia and myositis | 29/3,324 | 0.9% | 25/2,050 | 1.2% | 54/5,374 | 1.0% |
| Rhabdomyolysis and toxic myopathies | 0/3,455 | 0.0% | 0/2,111 | 0.0% | 0/5,566 | 0.0% |
| Liver disease | 0/3,438 | 0.3% | 5/2,107 | 0.2% | 14/5,545 | 0.2% |
| Cardiovascular disease | 72/3,455 | 2.1% | 69/2,113 | 3.3% | 141/5,568 | 2.5% |
| Mortality | 84/3,455 | 2.4% | 68/2,113 | 3.2% | 152/5,568 | 2.7% |
