# Supplementary material for: Regression discontinuity analysis for pharmacovigilance: statin example reflected trial findings showing little evidence of harm
Source: J Clin Epidemiol. 2022 Jan;141:121–31. doi: 10.1016/j.jclinepi.2021.10.003 (PMC8982642; doi:10.1016/j.jclinepi.2021.10.003)
Supplement: Supplementary file 5 [file mmc5.docx]

**Appendix E: Summary of results, RDA adjusted*, without outcomes occurring within two weeks or within 60 days of the index date, adhering practices**

| **Outcome** | **Without outcomes within two weeks of index date** | | **Without outcomes within 60 days of index date** | |
| --- | --- | --- | --- | --- |
|  | **MD/RD** | **95% CI** | **MD/RD** | **95% CI** |
| Type2 diabetes | 1.31 | -5.56 to 8.18 | -1.18 | -7.52 to 5.16 |
| Myalgia and myositis | 1.66 | -2.38 to 5.71 | 1.01 | -2.89 to 4.90 |
| Liver disease | 0.56 | -1.69 to 2.81 | 0.41 | -1.74 to 2.56 |
| Cardiovascular disease | 4.00 | -2.21 to 10.21 | 3.51 | -2.51 to 9.53 |
| Mortality | -3.63 | -10.06 to 2.80 | -4.39 | -10.71 to 1.93 |

*adjusted for age and sex
