# Supplementary material for: Regression discontinuity analysis for pharmacovigilance: statin example reflected trial findings showing little evidence of harm
Source: J Clin Epidemiol. 2022 Jan;141:121–31. doi: 10.1016/j.jclinepi.2021.10.003 (PMC8982642; doi:10.1016/j.jclinepi.2021.10.003)
Supplement: Supplementary file 6 [file mmc6.docx]

**Appendix F: Summary of results, RDA unadjusted and adjusted, simple linear regression, all practices**

| **Outcome** | **RDA** | | | | **Simple linear regression*** | |
| --- | --- | --- | --- | --- | --- | --- |
|  | **Unadjusted** | | **Adjusted*** | |  |  |
|  | **MD/RD** | **95% CI** | **MD/RD** | **95% CI** | **MD/RD** | **95% CI** |
| Total cholesterol | -1.47 | -2.16 to -0.78 | -1.37 | -2.05 to -0.70 | -0.84 | -0.90 to -0.79 |
| Type2 diabetes | 7.35 | -1.03 to 15.73 | 6.08 | -2.42 to 14.57 | 2.40 | 1.74 to 3.06 |
| Myalgia and myositis | 1.49 | -2.92 to 5.90 | 1.69 | -2.82 to 6.19 | 1.21 | 0.86 to 1.56 |
| Liver disease | 1.53 | -1.74 to 4.80 | 1.50 | -1.83 to 4.83 | -0.40 | -0.66 to -0.14 |
| Cardiovascular disease | 1.03 | -6.62 to 8.68 | 1.01 | -6.77 to 8.80 | 1.14 | 0.53 to 1.74 |
| Mortality | -1.02 | -8.68 to 6.64 | -0.72 | -8.52 to 7.07 | -0.97 | -1.58 to -0.36 |

*adjusted for age and sex
